# Are Virulence and Antibiotic Resistance Genes Linked? A Comprehensive Analysis of Bacterial Chromosomes and Plasmids

**DOI:** 10.3390/antibiotics11060706

**Published:** 2022-05-24

**Authors:** Helena Darmancier, Célia P. F. Domingues, João S. Rebelo, Ana Amaro, Francisco Dionísio, Joël Pothier, Octávio Serra, Teresa Nogueira

**Affiliations:** 1 Bacteriology and Mycology Laboratory, INIAV—National Institute for Agrarian and Veterinary Research, 2780-157 Oeiras, Portugal; helenadarmancier@gmail.com (H.D.); celiapfd@hotmail.com (C.P.F.D.); ana.amaro@iniav.pt (A.A.); 2 cE3c—Center for Ecology, Evolution and Environmental Change & CHANGE—Global Change and Sustainability Institute, Faculdade de Ciências, Universidade de Lisboa, 1749-016 Lisboa, Portugal; joaorebelo_4@hotmail.com (J.S.R.); dionisio@fc.ul.pt (F.D.); 3 Departamento de Biologia Vegetal, Faculdade de Ciências, Universidade de Lisboa, 1749-016 Lisboa, Portugal; 4 Atelier de Bioinformatique, ISYEB, UMR 7205 CNRS MNHN UPMC EPHE, Muséum National d’Histoire Naturelle, CP 50, 45 Rue Buffon, F-75005 Paris, France; joel.pothier@gmail.com; 5 INIAV—National Institute for Agrarian and Veterinary Research, Portuguese Plant Germoplasm Bank, 4700-859 Braga, Portugal; octavio.serra@iniav.pt

**Keywords:** antibiotic resistance, virulence, plasmid, Integrative and Conjugative Element, co-selection, genomics, evolution

## Abstract

Although pathogenic bacteria are the targets of antibiotics, these drugs also affect hundreds of commensal or mutualistic species. Moreover, the use of antibiotics is not only restricted to the treatment of infections but is also largely applied in agriculture and in prophylaxis. During this work, we tested the hypothesis that there is a correlation between the number and the genomic location of antibiotic resistance (AR) genes and virulence factor (VF) genes. We performed a comprehensive study of 16,632 reference bacterial genomes in which we identified and counted all orthologues of AR and VF genes in each of the locations: chromosomes, plasmids, or in both locations of the same genome. We found that, on a global scale, no correlation emerges. However, some categories of AR and VF genes co-occur preferentially, and in the mobilome, which supports the hypothesis that some bacterial pathogens are under selective pressure to be resistant to specific antibiotics, a fact that can jeopardize antimicrobial therapy for some human-threatening diseases.

## 1. Introduction

Antimicrobial resistance is a natural phenomenon, yet its emergence has been driven by antimicrobial exposure in healthcare, agriculture, veterinary settings, and the environment [1]. In the case of antibiotic use to cure bacterial infections, the onset of the disease dictates the prescription [2] and so antibiotics are specifically targeted to the bacterial pathogen. Recently, a study referred to a large increase in the prevalence of carbapenem-resistant *Klebsiella pneumoniae* and hypervirulent *K. pneumoniae*, revealing a worrying increase in this association between antibiotic resistance and virulence in human pathogens [3,4,5]. Another recent study also showed that 31% of 56 clinical isolates of *Pseudomonas aeruginosa* (an important agent of nosocomial infections worldwide), collected from different medical centers in Kenya between 2015 and 2020, harbor both virulence genes and a multidrug resistance phenotype. The authors report novel strains with extensive antimicrobial resistance genes (hereafter named AR) and the highest number of virulence factor genes (hereafter named VF) [6,7,8]. Therefore, natural selection might have been favoring the co-localization of virulence and resistance genes in the same cell genome or even in the same replicon (plasmid or chromosome). However, even when targeting pathogens, antibiotics also strike hundreds of commensal or mutualistic bacterial species comprising the trillions of cells present in the human or animal bodies.

Besides their therapeutic use, antibiotics have been used in medicine and veterinary care for decades, for prophylactic and metaphylactic uses [9]. In agriculture and animal production systems, for example, antibiotics are used on a very large scale, driven by economic interests [10]. In addition, there has been the reported release of antibiotics and active antibiotic residues into the environment, issuing from agriculture and the pharmaceutical industry [11]. This widespread and large-scale use has made antibiotics an emerging environmental contaminant [12]. Sewage waters issuing from urban areas, hospitals, and agricultural fields reach rivers [13] and contaminate agricultural fields. In this scenario, antibiotic resistance can be easily transferred between the environment, humans, and animals, as envisaged in the one health approach [1]. Thus, in the environment, all bacteria could potentially be equally exposed to these antibiotics, regardless of whether they are a human or an animal pathogen. However, as is the case for antibiotic exposure in infection therapy, environmental contamination from antibiotic use in agriculture can also be biased towards some antibiotics due to usage guidelines and regulations in place. 

Previous research has shown that the diversity of virulence and antibiotic resistance genes correlate positively across human gut microbial communities and their genomes (metagenomes) [14]. In other words, metagenomes with a higher diversity of drug resistance genes are also the ones with a higher diversity of virulence genes, and vice versa—metagenomes with a lower diversity of drug resistance genes are also the ones with a lower diversity of virulence genes [15]. A previous work has shown that this positive correlation arises whenever the transmission probability of bacteria, along with their genes, between people is higher than the probability of losing resistance genes. This is a likely relationship because fitness costs imposed by resistance determinants often disappear in a few tens or hundreds of generations after a new gene has arisen [16,17,18]. Therefore, the positive correlation did not arise due to co-selection by antibiotics on both gene types in the metagenome [15]. Human beings interact and exchange bacteria and resistance and virulence genes through physical contact—perhaps unexpectedly, individuals with a higher diversity of both gene types are the ones that have not taken antibiotics for a long time. Those individuals who have recently used antibiotics show a decreased diversity in these genes [15]. However, these works do not elucidate what to expect at the level of individual bacterial cells or replicon localization.

Recent studies on the co-localization of ARs and VFs have focused on the dynamics of antibiotic resistance in bacterial pathogens and its comparison with non-pathogenic bacteria [19,20]. It is particularly important, however, to understand the co-evolutionary paths of antibiotic resistance and bacterial virulence at a bacterial genome level, though, to evaluate potentially critical combinations that could hinder the treatment of human and animal bacterial infections. During this study, we tested the co-selection hypothesis at different genomic locations in an exceptionally large and comprehensive database and identified the most favored and the most disadvantaged combinations of AR and VF. It is an innovative work because it generates knowledge that allows the study of evolutionary forces and those driving the co-mobilization of every AR and VF type or class, in bacterial genomes, on a very large scale.

We consider the bacterial genome as two types of molecular replicon: the chromosome and the plasmids. Most of the housekeeping genes are chromosomally encoded. Plasmids, however, are especially important mobile genetic elements, part of the accessory genome responsible for local adaptation. They can encode for supplementary biochemical pathways, which allow the expression of the different phenotypes and lifestyles, adaptative traits enabling cells to respond to local competitive or environmental pressures, such as exposure to antibiotics, bacteriocins, heavy metals, or other xenobiotics. Plasmids are enriched with genes encoding extracellular traits, and for proteins targeted to the cell envelope, such as those in the bacterial secretion systems [21]. 

Plasmids can be mobilized from one bacterium cell into another and can carry Integrative Conjugative Elements (ICE), which are mobile genetic elements able to both integrate bacterial chromosomes by site-specific recombination or exist as autonomous plasmid-like conjugative elements [22]. ICEs are also known as conjugative transposons as they encode for the type IV secretion system (which are also bacterial virulence factors) that is necessary for horizontal gene transfer between cells. Moreover, ICEs can carry insertion sequences and/or transposons, Integrases, and the Relaxase enzyme, which is critical for conjugation [23]. 

However, as they also stay in the chromosome, we are considering here all the genes that can be on either type of replicon, such as ICE, for example, in a different category named ‘both’. It is believed that they mostly remain integrated into the chromosome, where they are transferred vertically. Nevertheless, under a lethal challenge, they can be horizontally transferred when the host cells are subject to DNA damage and SOS stress or under a starvation/stationary phase [23], in a small fraction of the bacterial population. ICEs can encode for adaptative niche traits as they harbor cargo genes conferring the following phenotypes: antibiotic resistance, the ability to metabolize a new carbon source, pathogenicity, or symbiosis islands [23].

The aim of this work is to understand whether evolution may have shaped bacterial genomes favoring a genetic link between virulence factors and antibiotic resistance-encoding genes, and to understand the overly complex relation between VF and AR at the genomic level.

## 2. Results

To try to capture evolutionary patterns on an overly broad scale, we retrieved all 16,632 complete closed bacterial genomes from the NCBI RefSeq database and classified all their replicons as chromosomal or plasmid. An additional genomic category was considered that included genes that have copies in the chromosome and in a plasmid of the same genome, and which could potentially be ICE. Each gene can only belong to one of these three categories—chromosome, plasmid, or ‘both’—as every gene that appears on both chromosome and plasmid is only considered in the category ‘both’. Note that a single cell may harbor more than one plasmid; therefore, even if two genes co-localize in plasmids, they may localize in different plasmids.

### 2.1. Genomic Organization of Antibiotic Resistance-Encoding Genes in Bacterial Genomes

To assess the identification and localization of antibiotic resistance-encoding genes within the bacterial genomes, we identified and classified all proteins from the dataset. To do so, we aligned all these protein sequences against antibiotic resistance databases: ResFinder, which consists of 3160 genes of acquired antibiotic resistance organized into 17 categories, including disinfectant resistance [24], and MUSTARD, which is a catalogue of 3.9 million proteins from the human gut microbiome organized into 41 categories [25]. For each genome, we identified all orthologs of each antibiotic resistance category, in each replicon type: chromosome or plasmid. We then counted the number of AR orthologues of each category that are located only in the chromosome, only in their plasmids, or simultaneously in both replicons. One may expect that larger plasmids or chromosomes would contain more AR orthologues. To remove this effect, we proceeded by dividing the counts by the total number of coding sequences (CDS) that are in each of the genomic locations: on the chromosome only, on the plasmids only, or on both locations in the same genome (57020371, 1531377, and 29622, respectively).

**Figure 1:**
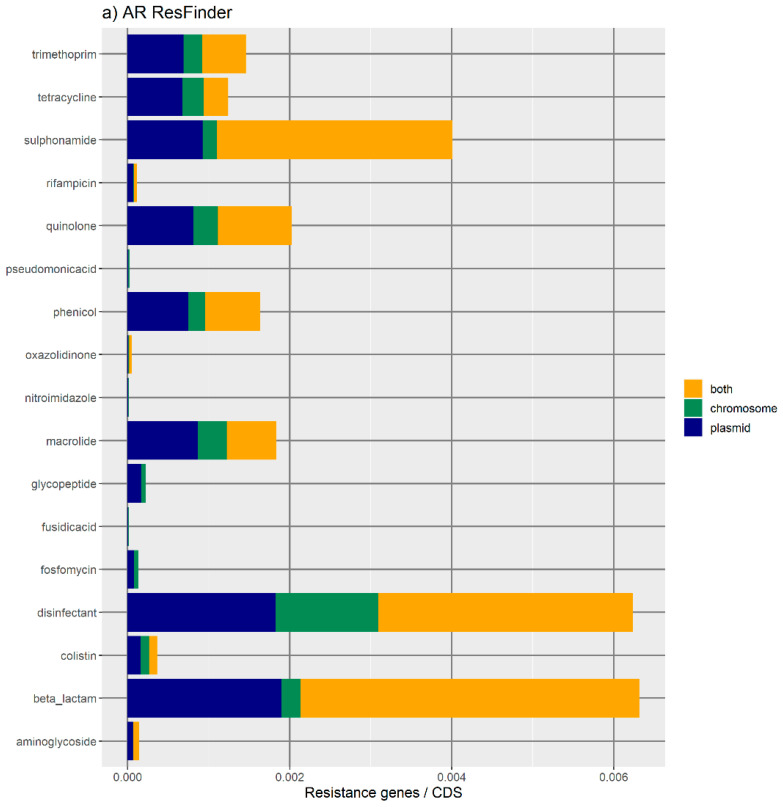

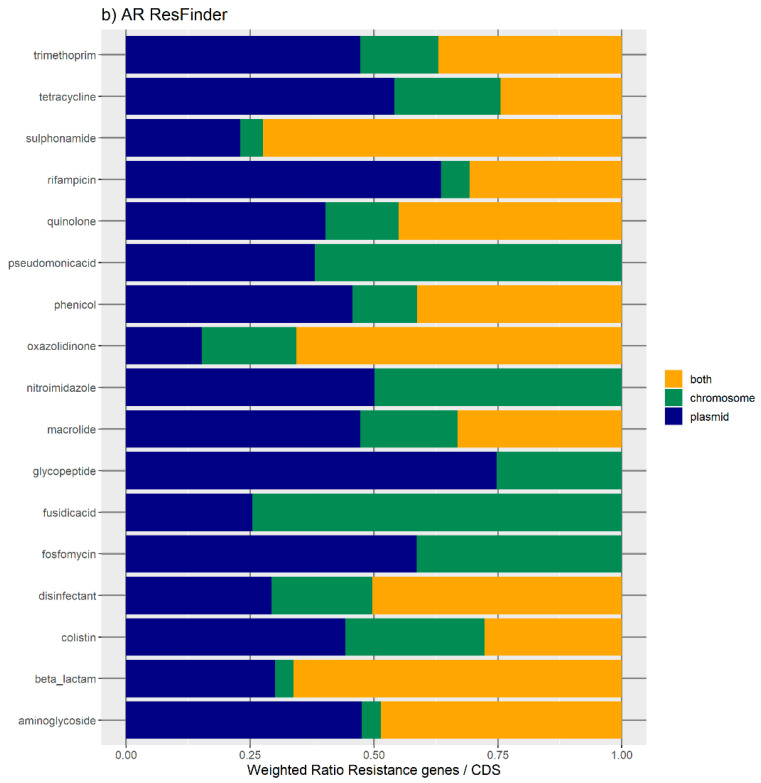

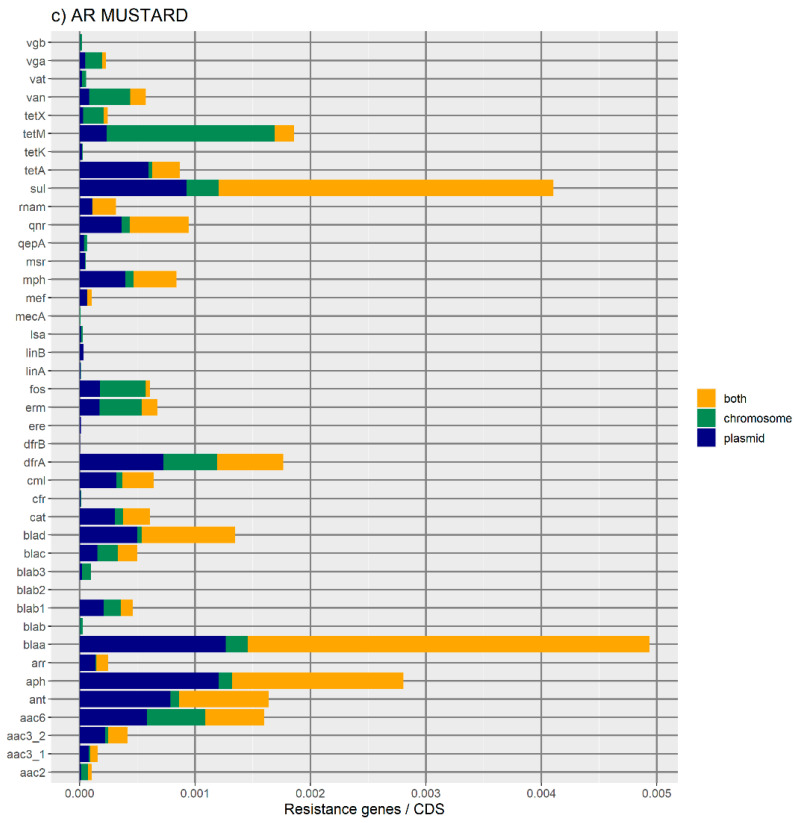

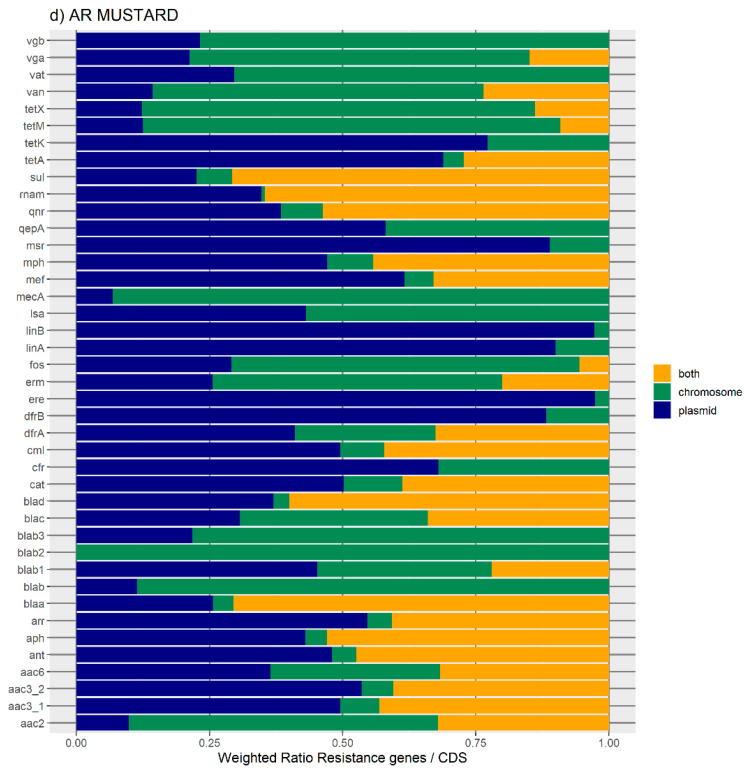
Bar plots and weighted bar plots of the number of antibiotic resistance orthologues. Because replicons vary in size, the number of resistance orthologues in each genome was divided by the amount of coding DNA sequences (CDS) in that genome (in that plasmid, in that chromosome, or in both). (**a**,**b**) Data from the ResFinder database; (**c**,**d**) data from the MUSTARD database. (**a**) For the ResFinder orthologues, the highest amount of antibiotic resistance orthologues is in the ‘both’ location (yellow). Beta-lactamase, sulphonamides, and disinfectant orthologues are more often (more than 50%) located in both replicons than in only a plasmid or the chromosome. (**b**) For the ResFinder orthologues, weighted bar plot shows that there are no fosfomicin, nitroimidazole, pseudomonicacid, fusidic acid, or glycopeptide orthologues in the ‘both’ location (yellow). Orthologues located in chromosomes (green) have a lower proportion compared to the other two genomic locations. (**c**) For the MUSTARD orthologues, there is a higher number of antibiotic resistance orthologues located simultaneously in both a plasmid and the chromosome (‘both’ location in yellow). There is a higher amount of *blaa*, *rnam*, and *sul* and orthologues. (**d**) For the MUSTARD database, there is a higher proportion of orthologues located in plasmids (blue). There is a lower proportion of orthologues located in ‘both’ a plasmid and the chromosome (yellow) than in a plasmid only or in the chromosome only. Moreover, 15 out of 45 orthologues (e.g., the orthologues of *vgb*, *vat*, or *tetk*) are present only in a plasmid or in the chromosome (i.e., without a ‘both’ location).

Figure 1 represents the distribution of the normalized number of antibiotic resistance gene orthologues per antibiotic class. According to ResFinder (Figure 1a,b), the largest amount of ARs belong to the category of beta-lactamase, sulphonamide, quinolone, macrolide, phenicol, trimethoprim, and tetracycline, which are present in the chromosome, plasmids, or in both chromosomes and plasmids belonging to the same bacterial genome. We also found large amounts of disinfectant-resistant genes. Of note, no fosfomycin, nitroimidazole, pseudomonicacid, or glycopeptide orthologues were found in the ‘both’ location. Interestingly, most orthologues for disinfectant resistance also appear to be preferentially encoded in the bacterial mobilome (here represented by the plasmids and ICE). Figure 1b represents the relative number of AR orthologues per genomic location. 

Overall, the results obtained with the two databases showed a high number of antibiotic resistance orthologues located in both genomic locations of the same genome, suggesting that ARs are carried in the bacterial mobilome. AR genes encoding beta-lactamases showed the highest numbers of orthologues in both the ResFinder and MUSTARD databases. This latter showed that the *blaa* category encodes Class A beta-lactamases, which are the predominant beta-lactamases found in the bacterial genome. All beta-lactamases can be found in plasmids and both plasmids and chromosome, except subclass B2 beta-lactamases, which are found only in the chromosome.

**Figure 2:**
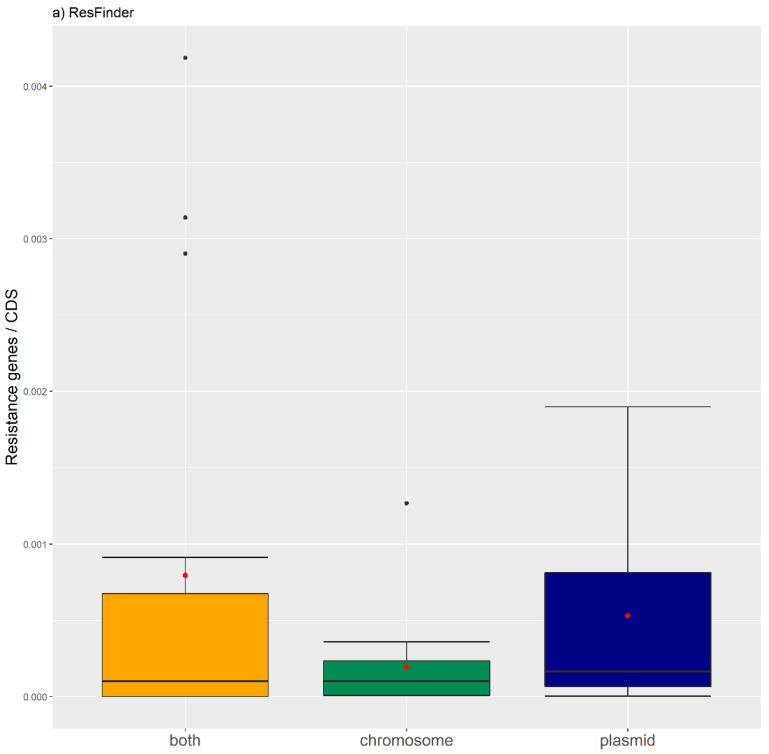

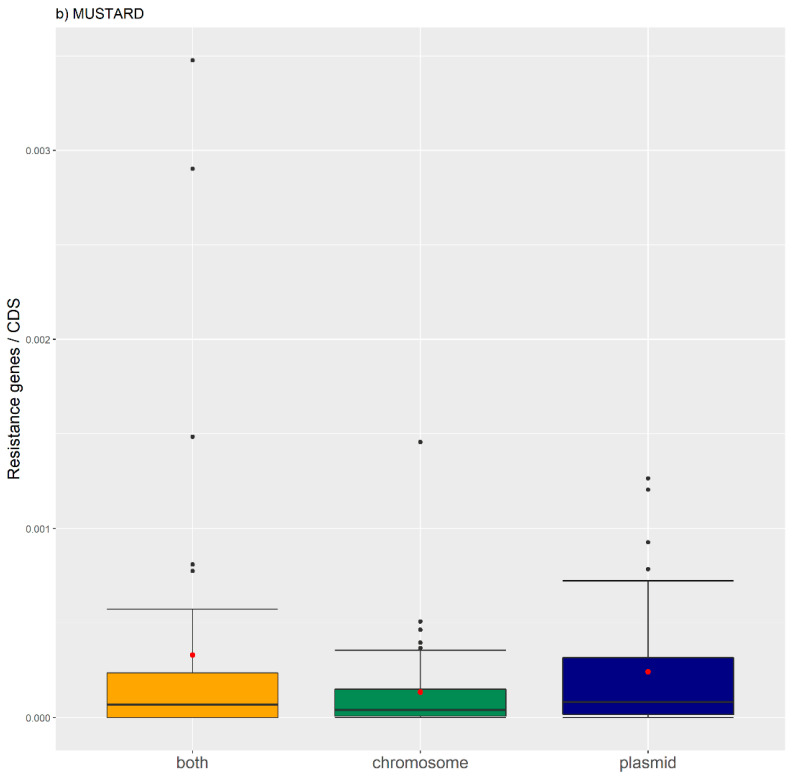
Boxplots of the antibiotic resistance orthologues per genomic location. (**a**) ResFinder orthologues. (**b**) MUSTARD orthologues. The red dot represents the mean.

We have also generated boxplots of the antibiotic resistance orthologues per CDS for each genomic location (Figure 2). The boxplots do not show significant differences in the relative frequencies of ARs between the different genomic locations. The Kruskal–Wallis test was conducted to examine the differences between the relative number of antibiotic resistance orthologues according to the three types of genomic location. No significant differences were found (ResFinder: Chi square = 50.00, *p*-value = 0.2815, df = 16; and MUSTARD: Chi square = 116.42, *p*-value = 0.05838, df = 40).

### 2.2. Genomic Organization of Virulence Factor-Encoding Genes in Bacterial Genomes

We have also computed the number of virulence factor orthologues as described in the previous section. We calculated the number of orthologues of virulence factors (VF) grouped into various categories of the VFDB database, and according to their genomic location: only in the chromosome, only in a plasmid, or in both simultaneously. Once again, we may expect that larger plasmids or chromosomes would contain more VF orthologues. To remove this effect, we proceeded by dividing the counts by the total number of CDS in each of the genomic locations, as we did for the analysis of the ARs.

**Figure 3:**
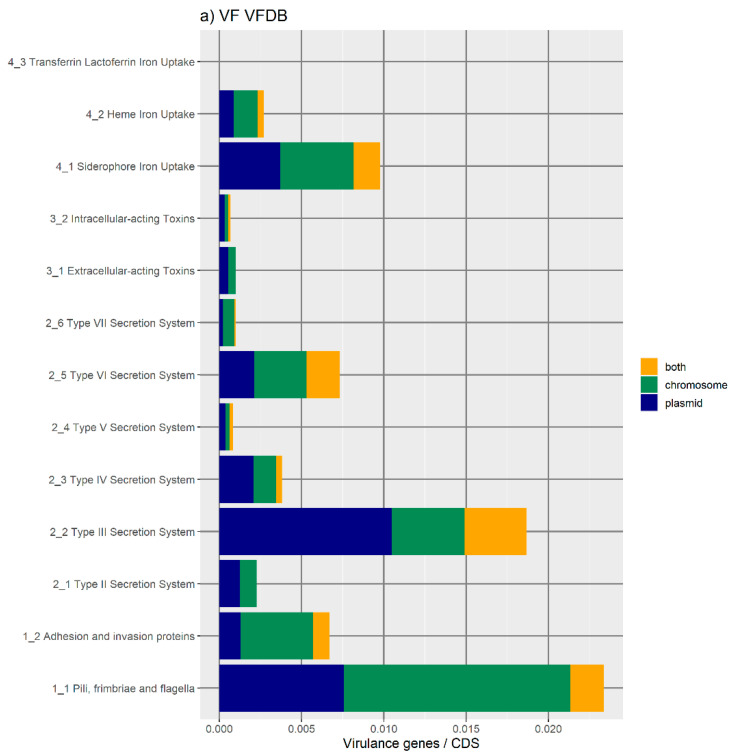

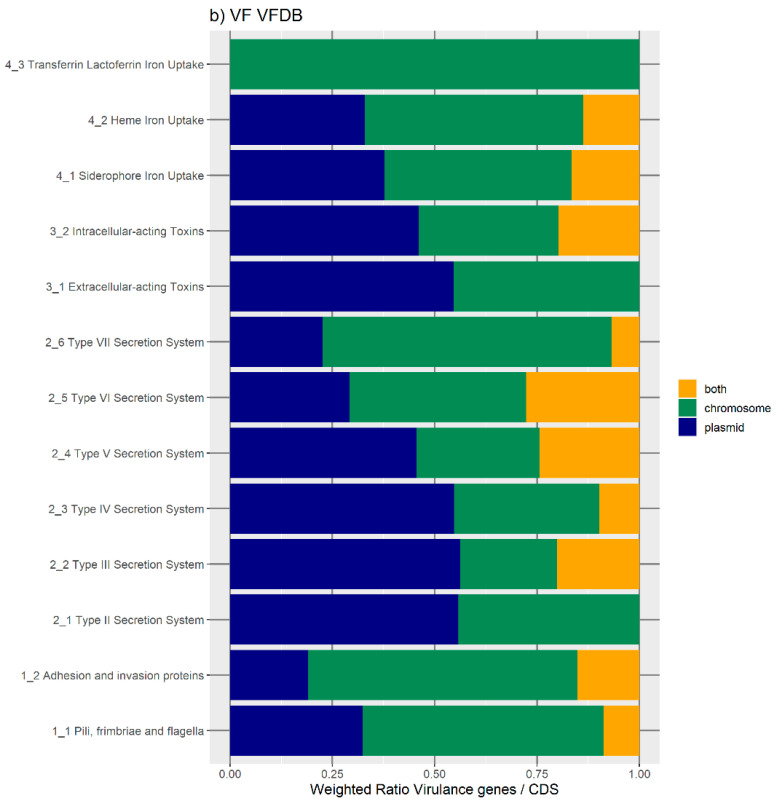
Bar plots (**a**) and weighted bar plots (**b**) of the number virulence factor orthologues in VFDB. Because replicons vary in size, the number of virulence orthologues in each genome was divided by the amount of coding DNA sequences (CDS) in that genome (in that plasmid, in that chromosome, or in both). (**a**) There is a higher number of virulence factor orthologues located in chromosomes (green) than only in a plasmid or in both a plasmid and the chromosome. There is a higher number of orthologues of Pili, fimbriae, and flagella and type III secretion systems than orthologues of the other virulence genes. (**b**) Orthologues of virulence genes are proportionally more present in the chromosome (green) only or in a plasmid only (blue). Transferrin and lactoferrin iron uptake orthologues are only located in chromosomes (green). Extracellular acting toxins and type III secretion system orthologues were either located in a plasmid (blue) or in the chromosome (green), not in both (yellow).

**Figure 4:**
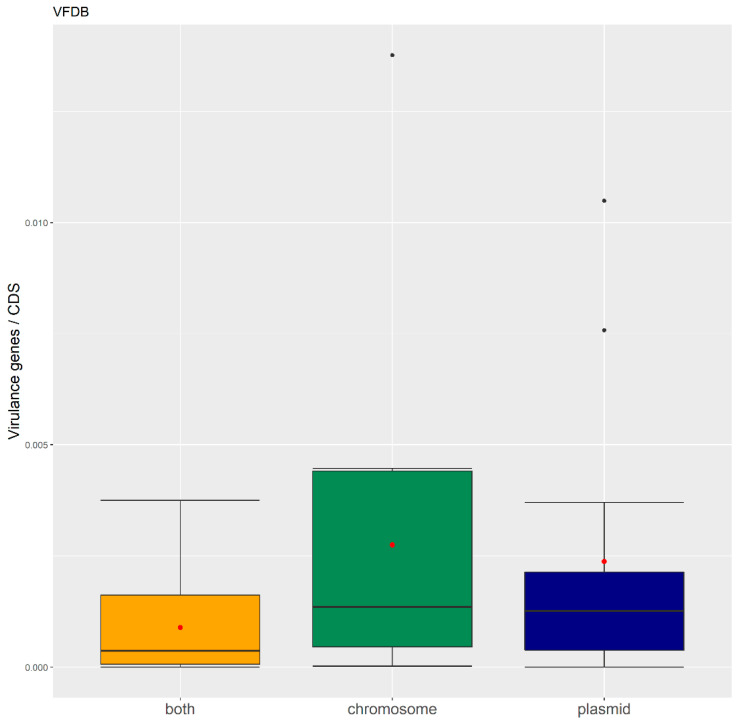
Boxplots of virulence factor orthologues per genomic location. The variance is higher in chromosomes (green). Orthologues found in plasmid (blue) and both (yellow) locations have similar variation. The red dot represents the mean.

Although there are many virulence genes encoded on the plasmid, the chromosomal location is also very frequent in most bacteria (Figure 3 and Figure 4). The results shown in Figure 3 show an uneven distribution of the various categories of virulence factors in each genome location. Pili, fimbriae, and flagella are the most representative virulence factors encoded on the chromosomes (Figure 3a). They all belong to the adhesion and invasion systems, which are essential to the colonization of the host or substrate, in the case of environmental species.

Another important category of virulence factor is the type III secretion system (T3SS) [26], which, on the other hand, appears to be mainly encoded on plasmids (Figure 3a). They are important for the interaction of the cell with the host and are involved in the secretion of bacterial toxins directly into the host cell [27]. 

Regarding iron uptake systems, there is an uneven distribution according to the various categories: siderophore, heme, transferrin, and lactoferrin-mediated iron uptake systems. Siderophores are the most abundant, followed by heme-mediated iron uptake proteins, while orthologues of transferrin- and lactoferrin-mediated iron uptake proteins appear to be rare and chromosomally encoded (Figure 3a).

We can also see elevated levels of type IV and type VI secretion systems (T4SS and T6SS) [28,29] that, together with T3SS, are responsible for cell-to-cell interactions, such as the injection of toxins into host cells, or in conjugation and quorum sensing in bacteria. All these coding traits can be located on the chromosome, on plasmids, or present in both locations on the same genome (Figure 3a). 

T4SS is part of the conjugation systems that are put in place to allow the transfer of mobilizable plasmids and conjugators as well as ICE from one cell to another [30]. The majority of the T4SS share a plasmid location.

We have also generated boxplots (Figure 4) and conducted the Kruskal–Wallis test to analyze the differences between the relative number of virulence gene orthologues according to the three types of genomic location. No significant differences were found (Chi square = 33.615, *p*-value = 0.4375, df = 12).

### 2.3. Correlation Analysis of Antibiotic Resistance- and Virulence Trait-Encoding Genes Per Genomic Location

**Figure 5:**
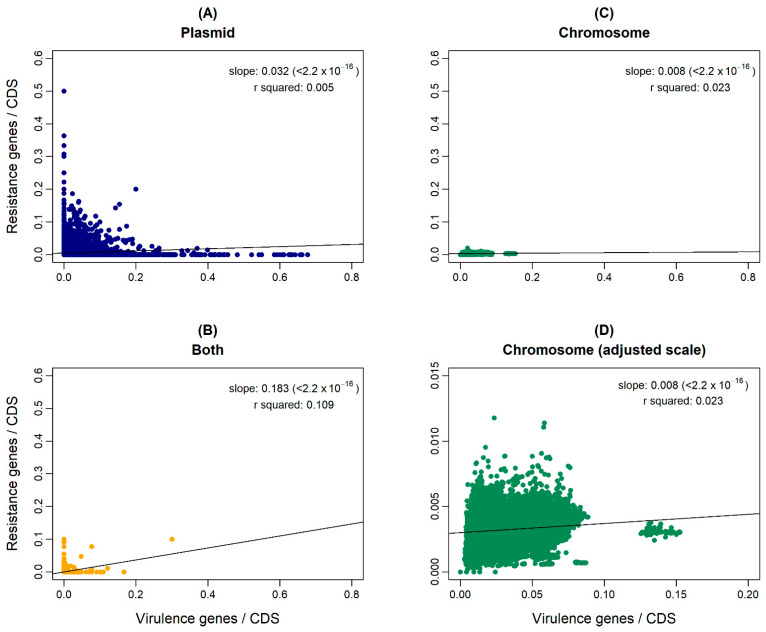
Relationship between antibiotic resistance gene orthologues of the ResFinder database (vertical axes) and virulence gene orthologues of the VFDB (horizontal axes). To correct for the size of the corresponding locations, the number of resistance and virulence genes in each genome was divided by the amount of coding DNA sequences (CDS) in that genomic location. Each point represents a genome. The colors indicate the genomic location: plasmid (blue), chromosome (green), or ‘both’ (yellow). (**C**,**D**) show the same data; however, (**D**) has the scale adjusted to better demonstrate the distribution of points. In (**A**), plasmid location (R = 0.005, slope = 0.032, *p*-value = ~0). In (**B**), both locations (R = 0.109, slope = 0.183, *p*-value = ~0). In (**C**,**D**), chromosome location (R = 0.023, slope = 0.008, *p*-value = ~0).

The hypothesis that antibiotic resistance and virulence are genetically linked stems from the fact that, in human and veterinary medicine, the use of antibiotics, when not for prophylaxis, is adopted specifically to treat a bacterial infection, i.e., antibiotic therapy is targeted at pathogenic bacteria. If this assumption holds, we expect these two traits to be co-selected and mobilized together in plasmids—for example, in microbial communities [14]. Thus, first, we decided to assess the relationship between the number of ARs and VFs in each genome, at each genomic location, following a similar algorithm as in the previous sections. We assigned each orthologue to a genomic location: only on the chromosome, only on the plasmid, or both on the chromosome and plasmid. Then, we counted, for each genome, the number of ARs and VFs in each location. Finally, we divided these counts by the number of coding DNA sequences (CDS) in each location. These new values were used to generate the linear model in Figure 5. In general, there are no correlations between the number of ARs and VFs in bacterial genomes (Figure 5).

For the case of genes that are present both in the chromosome and in a plasmid, the sample size is much smaller than for the other locations. Furthermore, the size of a possible ICE-like mobile genetic element is expected to be even smaller than that of plasmids and chromosomes, which makes it risky to make direct comparisons. This smaller size makes the co-occurrence of ARs and VFs less likely and may explain the vertical and horizontal clustering of points seen in the ‘both’ panel of Figure 5B.

**Figure 6:**
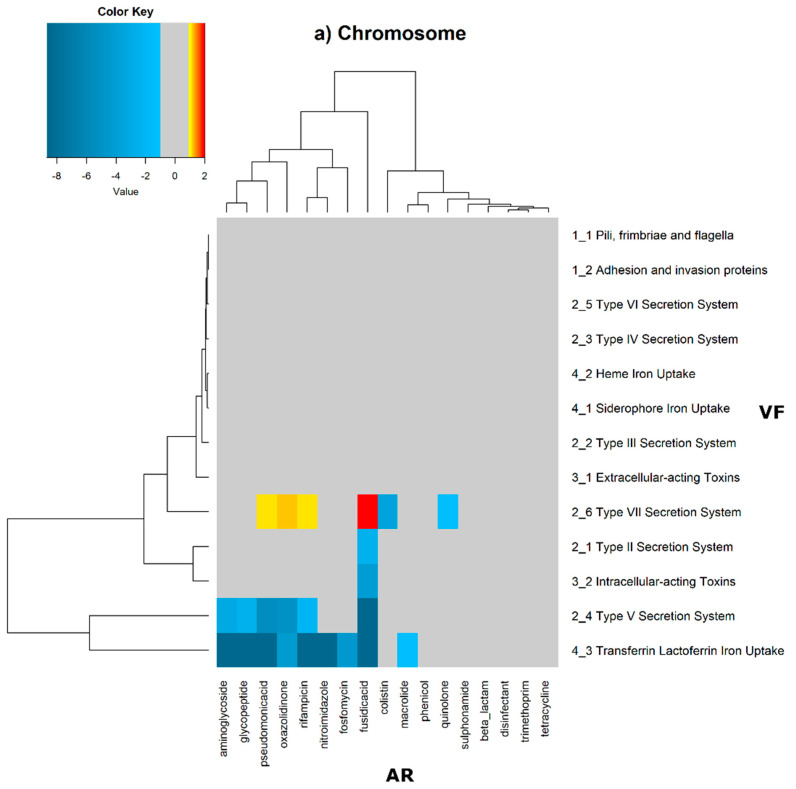

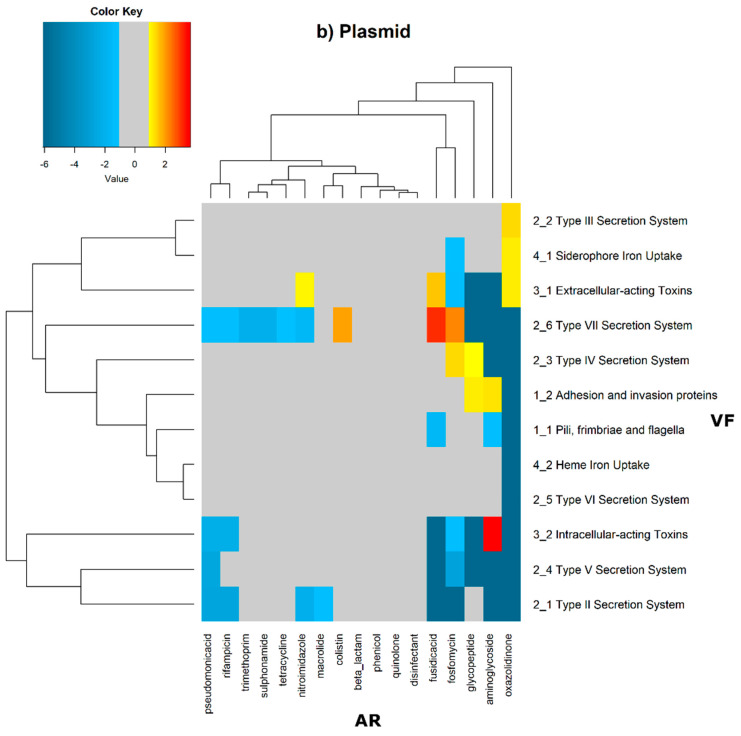

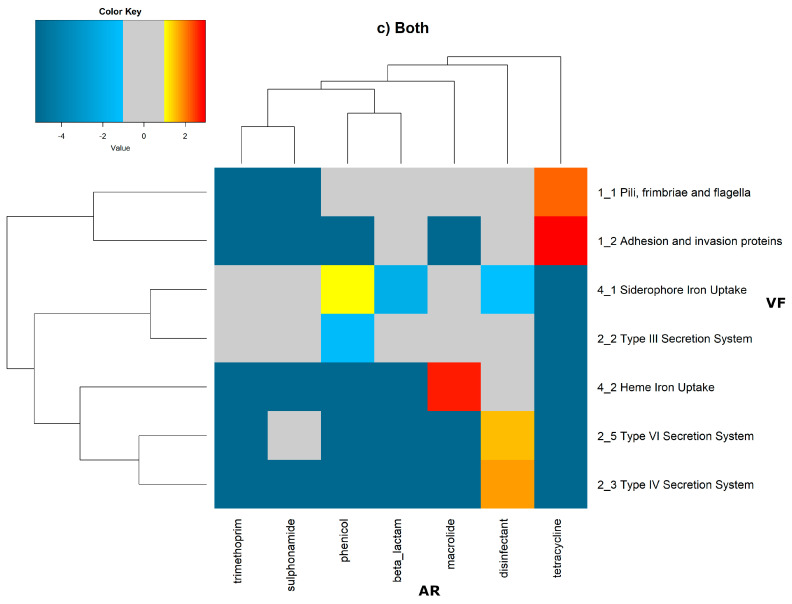
Heatmaps of the number of antibiotic resistance orthologues of the ResFinder database (*x* axis) and virulence factor orthologues of the VFDB database (*y* axis) in each genomic position. The values correspond to the Log_2_ of the quotient of observed/expected values. The yellow to red shades represent a higher-than-expected number of co-occurrences; the blue shades represent a lower-than-expected number of co-occurrences; and the grey represents values close to the expected values. (**a**) The higher number of ResFinder’s orthologous proteins that co-occurred with VFDB’s orthologous in the chromosome is between type VII secretion systems and fusidic acid. (**b**) In plasmids, the highest number of co-occurrences is between type VII secretion systems and fusidic acid and between intracellular acting proteins and aminoglycosides. (**c**) The number of co-occurrences in both the chromosome and plasmid is low. Consequently, we detected no co-occurrences involving six out of thirteen orthologues of virulence genes. There is a high number of co-occurrences of disinfectant orthologues with type IV and type VI secretion systems. The highest number of co-occurrences is between adhesion and invasion proteins and tetracyclines and between heme-mediated iron uptake and macrolides.

**Figure 7:**
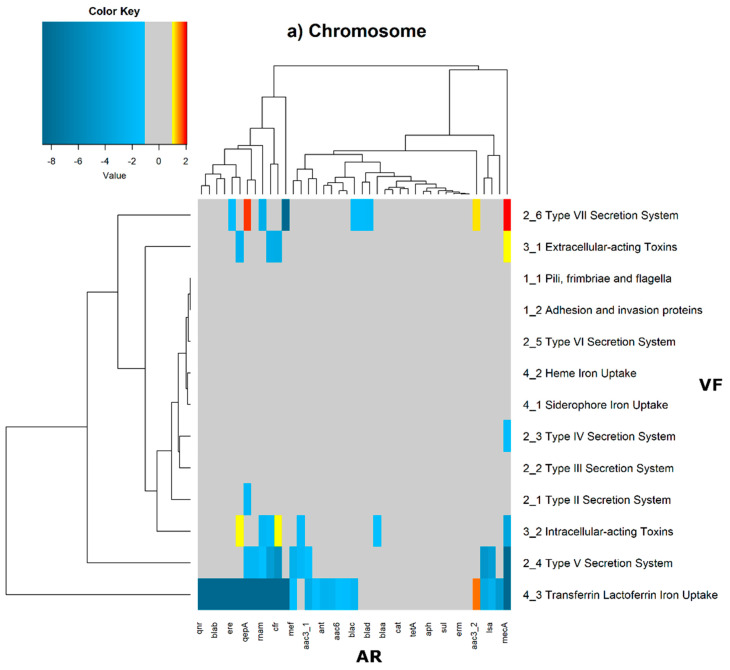

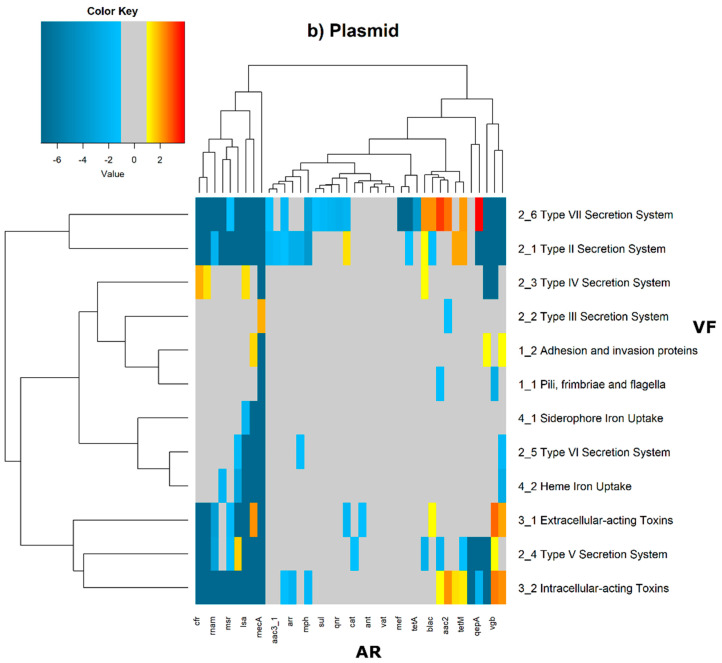

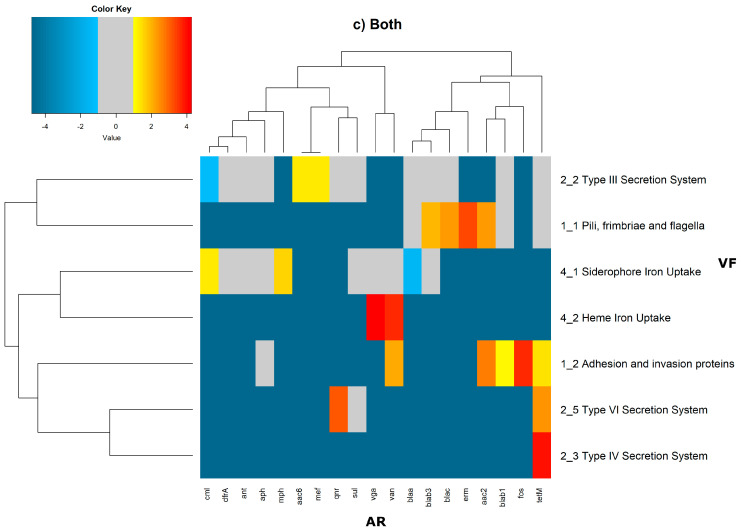
Heatmaps of the number of antibiotic resistance orthologues of the MUSTARD database (*x* axis) and virulence factor orthologues of the VFDB database (*y* axis) in each genomic position. The values correspond to the Log_2_ of the quotient of observed/expected values. The yellow to red shades represent a higher-than-expected number of co-occurrences; the blue shades represent a lower-than-expected number of co-occurrences; and the grey represents values close to the expected values. (**a**) The highest number of co-occurrences of proteins orthologous to the MUSTARD database is between type VII secretion systems and the *mecA* antibiotic resistance gene, and between type VII secretion systems and *aac2*. (**b**) In plasmids, the highest number of co-occurrences appear to involve type VII secretion systems and toxins. (**c**) The ‘both’ genomic position has the lower number of co-occurrences; there is a more diverse pattern of co-occurrences. We observed no co-occurrence involving six out of thirteen orthologues of virulence genes.

In order to evaluate the distribution of the co-occurrence of each of the antibiotic resistance gene categories and the virulence gene categories in the genomes used in this study, we constructed a contingency table. Each field of the contingency table contains the number of genomic elements containing orthologues from each of the AR and VF categories. These counts were made for all the AR and VF categories’ combinations. Next, we generated a matrix containing the expected values of counts for each of the previous combinations. They are the product of the orthologous counts of each of the AR and VF categories in all genomes, divided by the total number of counts. Finally, we have computed the Log_2_ of the quotient of each observed value by the respective expected value. Heatmaps, as represented in Figure 6 and Figure 7, were generated for the co-occurrences between all classes of ARs and VFs. Values in each position of the matrix that are greater than zero represent the genomes for which there are a higher number of co-occurrences between ARs from a given category and VFs from another category than expected at random and may mean that there is co-selection of these two genetic determinants. Conversely, a value below zero means that co-occurrence between a given AR category and another given category of VF is rarer than what would be expected to happen by chance, perhaps due to counter-selection. However, we have only considered as relevant those that are above 1 (represented in the yellow to red color shades) or below −1 (represented in blue shades). For these calculations, we have considered that each protein of the dataset can be classified into different AR or VF categories. With this choice, we are assuming that the same protein can belong to different antibiotic resistance mechanisms. An example of this is proteins that are part of the efflux pumps that perform the nonspecific extrusion of antibiotics belonging to different classes.

The co-occurrences that vary most according to genomic location involve T7SS, which frequently co-occur with fusidic acid resistance genes, either on the chromosome or on the plasmid, but never on both simultaneously (Figure 6 and Figure 7).

On the chromosome, fusidic acid resistance genes, on the other hand, do not occur with many different categories of other antibiotic resistance genes, such as colistin. At this location, apart from genes encoding transferrin-mediated iron uptake (for which there are no co-occurrences at any genomic locus), several other co-occurrences are exceedingly rare, as is the case for T5SS, with several distinct categories of antibiotic resistance genes. 

In plasmids, there is a strong association between genes encoding for intracellularly acting toxins and those for aminoglycoside resistance, and oxazolidinone resistance genes co-occur frequently with some virulence factor genes, such as T3SS, the siderophore iron uptake system, or extracellularly acting proteins, while it co-occurs less than expected with all other virulence factors.

It is also worth noting that the heme-mediated iron uptake-encoding traits tend to co-occur with those for macrolide resistance at the ‘both’ loci. Transferrin-mediated iron uptake-coding genes only co-occur frequently with antibiotic resistance genes on the chromosome. The results in Figure 6 also highlight that adhesion and invasion virulence factor-encoding genes often co-occur with tetracyclines only at the ‘both’ loci, and with aminoglycosides and glycopeptides at the plasmid site, but to a lesser extent.

## 3. Discussion

This work aimed to study the evolutionary genomic dynamics of antibiotic resistance and virulence in bacteria. To have large-scale data and to perform a comprehensive study, we collected 16,632 reference genomes in the RefSeq database to address the correlation between the genomic locations of genes encoding for antibiotic resistance and bacterial virulence at two different scales. We first observed that the number of AR orthologues of all categories taken together does not differ significantly between the three locations (on the chromosome only, on a plasmid only, or on both). We reached the same conclusion for orthologues of VFs. In a second approach, which is novel, we analyzed the co-occurrences of each AR category with each VF category, by genomic location. This latter approach revealed co-occurrence patterns that are worth being addressed in more detail, and this will pave the way for further studies. 

For this study, we have used the dataset of bacterial genomes from RefSeq, the NCBI Reference Sequence database, which consists of a non-redundant, well-annotated set of reference sequences including genomic and protein sequences. This dataset, however, does not represent a random sample of bacterial genomes. In fact, we expect this collection to be skewed towards bacterial genomes of human and medical interest. In this sense, an overrepresentation of human pathogens would explain the overrepresentation of VFs that we have found.

The asymmetrical distribution per replicon of the ARs and VFs can result from the fact that we have 37-fold more coding sequences (CDS) at the chromosomal location than the plasmid one, and 51-fold more CDS in plasmids than in ‘both’ locations. This is also because we have as twice as many chromosomes as plasmids on our dataset, but also because most of the plasmids are indicated to encode around 60 proteins and are thus much smaller than chromosomes [30], and ICE are even smaller than plasmids. 

The fact that a particular gene is found on both the chromosome and a plasmid may indicate that this gene is in mobile regions of the chromosome, namely in ICE. According to Johnson and Grossman [23], antibiotic resistance genes mobilized from cell to cell by conjugative plasmids tend to move transiently or permanently onto the chromosome and do not remain in the cell as a stable plasmid. Therefore, one would expect that the antibiotic resistance genes found to have both a chromosome and plasmid location would be those clustered in the ICE and, therefore, also belong to the mobilome (the collection of mobile genetic elements).

### 3.1. The Antibiotic Resistance Genes Located in Plasmids Are Involved in Bacterial Local Adaptation

The normalization of the number of ARs by the number of CDS allowed the observation that there are many antibiotic resistance genes that are encoded in mobile regions of the genome, such as the ‘both’ category and in plasmids. This observation suggests that antibiotic resistance is a volatile and evolving trait, easily gained and lost, reinforcing the idea that these genetic elements are drivers of bacterial adaptation to the environment.

In this study, plasmids appear to be enriched with the following antibiotic resistance mechanisms: nucleotidyltransferase, beta-lactamase, phosphotransferase, and acetyltransferase. The production of hydrolyzing enzymes such as beta-lactamases encoded by *bla* genes is among the most important mechanisms for beta-lactams [31]. Bacteria encoding beta-lactamases are able to detoxify the local environment, hence rescuing nearby sensitive bacteria [32]. Other important enzymes include family members of nucleotidyltransferases (e.g., *ant* genes), phosphotransferase (e.g., *mph* and *fos* genes), and acetyltransferase (e.g., *aac* and *cat* genes), which confer resistance to aminoglycosides, macrolides, chloramphenicol, and fosfomycin, among others [33]. The link between antimicrobial use and resistance is complex, but the emergence of resistance is likely to be specific to each drug and to each microorganism [34]. Therefore, horizontal gene transfer and clonal expansion are giving rise to highly resistant microorganisms [5,35]. Nevertheless, other factors, such as cross-selection and co-selection, are highly pertinent while addressing antibiotic resistance [34]. 

We have also noticed that resistance to disinfectants is a very frequent trait, whose encoding genes preferentially belong to the ‘both’ category—that is, orthologues of disinfectant resistance are found in both the chromosome and a plasmid of the same cell. The link between disinfectants and antibiotics can be illustrated by the example of the use of quaternary ammonium compounds and sulphonamides since the 1930s, which has facilitated the spread of class 1 integrons and, thus, the evolution of antibiotic resistance in clinically relevant bacteria [36]. Given the fact that the use of disinfectants to clear pathogenic bacteria is recent, the results obtained here allow us to access the recent evolution of bacteria. The finding that the determinants of resistance to disinfectants are frequent and preferentially encoded in the mobile genome is a perfect example of the speed of the adaptive response and evolution of bacterial genomes in response to new environmental stresses. It has already been shown that biocidal agents used for disinfection can enhance antibiotic resistance in Gram-negative species [37]. Furthermore, in almost 90% of the outbreaks, the isolated pathogen turned out to be highly resistant to the disinfectant [36].

### 3.2. Mobile Genetic Elements Encode for Extracellular Traits and Cell-to-Cell Interactions

If we consider the considerable number of VFs that exist in the totality of genomes under study in this paper, we can observe that they are frequently encoded on the bacterial chromosome. This observation can reinforce the idea that many virulence traits are, in fact, essential to bacterial lifestyle and tend to be part of the core genome, rather than part of the arsenal for the colonization of new niches or environments. However, when we look at each of the VF categories individually, we can see that there are different patterns of genomic localization.

In this study, plasmids appear to be enriched with genes encoding extracellular traits, and for proteins targeted to the cell envelope, such as those pertaining to the bacterial secretion systems. Our previous results show that virulence factors that are targeted to the cell envelope or the outside medium are encoded in the mobilome [21]. This result seems coherent with the hypothesis that genes encoding for environmental adaptation and communication tend to be more mobile. 

ICE are characterized by encoding T4SS, the conjugation pilus that enables them to ‘jump’ from the bacterial chromosome into an autonomous plasmid-like replicon able to synthetize the conjugative pilus, a T4SS, and be transferred from one cell into another [23], which may explain the elevated levels of co-occurrence between the T4SS and resistance to disinfectants.

### 3.3. Co-Selection between Antibiotic Resistance and Bacterial Virulence at Genomic Level

The natural selection exerted by antibiotics administered in the context of infection is not only exerted on bacterial pathogens. The entire host’s microbiome will be under antibiotics’ selective pressure, which weakens the association between antibiotic resistance and virulence in bacteria.

If, however, in modern industrialized societies, the onset of a bacterial infection triggers compliance to a standardized therapeutic protocol for that bacterial infection, then we are reinforcing the idea that there is an association between certain pathogenicity mechanisms, characteristics of certain bacterial infections, and resistance to the antibiotics most used to treat that type of infection. On the other hand, approaching the question from the point of view of bacterial evolution, we can imagine that resistance to the antibiotics most described for a given infection will confer an adaptive advantage to the pathogenic clones. From this point of view, antibiotic resistance is seen as a virulence factor, and is, therefore, in the limit, part of the infection mechanism since it will be under selective pressure.

This large-scale study did not detect a strong correlation between antibiotic resistance and virulence in bacterial genomes, even though bacterial reference genomes were biased towards human bacterial pathogens. The widespread transmission of antibiotic resistance traits, together with the permanent exposure to ubiquitous antibiotics, also undermines the hypothesis of the correlation between antibiotic resistance and bacterial virulence, thus explaining the results.

The novelty of this study, however, comes from the extremely broad and comprehensive analysis that highlights which combinations of distinct categories of AR and VF are in genetic linkage, and which could potentially be epidemic-prone due to their location in a mobile genomic locus.

On chromosomes and in plasmids, there are some strong co-occurrences between fusidic acid resistance and type VII secretion systems (T7SS), suggesting that resistance to this type of antibiotic is an important feature. Fusidic acid is bacteriostatic that is widely used to treat skin infections by Gram-positive bacteria, and that is also active against tuberculosis [27,38]. T7SS plays a significant role in mycobacteria, and in the human pathogen *Mycobacterium tuberculosis*, the main causative agent of tuberculosis [39]. These strong co-occurrences in many genomes, and especially on the chromosome, may highlight the evolution of the bacterial genome in response to antibiotic therapy for tuberculosis.

T5SS is a widely distributed autotransporter system involved in the secretion of bacterial toxins of *Escherichia coli* into the surrounding extracellular milieu [40]. *E. coli* is a particularly important human and animal pathogen, and, so, this differential co-occurrence with different antibiotic resistance categories of genes may result from differential selection pressure exerted, for example, due to the therapeutic protocols to cure *E. coli* infections.

Co-occurrence analysis leverages an understanding of the genomic evolution of human and animal pathogens and will therefore foster our understanding of the selective pressures favoring the antibiotic resistance of major bacterial pathogens.

### 3.4. Conclusions

With this work, we aimed to test whether there would be an association between antibiotic resistance and bacterial virulence at the genomic level. To do this, we analyzed an exceptionally large set of reference bacterial genomes. Although the dataset studied here was expected to be enriched with pathogenic bacterial genomes, we did not identify an overall trend towards the existence of a genetic linkage between virulence and antibiotic resistance at either the chromosomal or plasmid level. However, there are patterns of association between some virulence categories and antimicrobial resistance factors at different genomic locations that point to the evolution of bacterial genomes dictated by antibiotic use.

## 4. Methods

### 4.1. Data Gathering and Preparation

As a first step to begin this large-scale analysis, 16,632 complete bacterial genomes were retrieved from the National Center for Biotechnology Information (NCBI) Reference Sequence (RefSeq) database, which provides a non-redundant collection of sequences representing genomic data, transcripts, and proteins, in fasta format. Our dataset is composed of protein sequence fasta format files derived from every complete bacterial genome present in the repository, downloaded via command line, from https://ftp.ncbi.nlm.nih.gov/genomes/refseq/bacteria/ (accessed on 19 October 2020).

### 4.2. Blast against Selected Databases

The BLAST+ executables package (ncbi-blast-2.9.0+ version) was downloaded from the NCBI website (ftp://ftp.ncbi.nlm.nih.gov/blast/executables/blast+ (accessed on 11 November 2019)) [41]. MUSTARD is a database of antimicrobial resistance determinants that was generated using a 3-dimensional modeling-based approach, called pairwise comparative modeling (PCM), that accurately predicts functions of proteins that are distantly related to proteins with known functions from the human intestinal microbiota. This database is structured in 41 categories. MUSTARD was downloaded from http://mgps.eu/Mustard/db/all_ard.zip (accessed on 26 October 2020) [25]. 

ResFinder is a comprehensive and up-to-date database of acquired genes and chromosomal mutations mediating antimicrobial resistance in total or partial DNA sequences of bacteria of major public health relevance. This database is divided into 17 categories. The ResFinder database was downloaded from https://bitbucket.org/genomicepidemiology/resfinder_db.git, accessed on 30 October 2020 [24].

The Virulence Factor Database (VFDB) is a collection of bacterial virulence factor protein families with current and in-depth coverage of the major virulence factors of the best-characterized bacterial pathogens. This database contains a total of 13 functionally classified fasta files of bacterial virulence factor protein sub-families. Files were downloaded from http://www.mgc.ac.cn/VFs/Down/VFDB_setB_pro.fas.gz, accessed on 6 November 2020 [42]. 

Protein sequence fasta files pertaining to the databases were formatted in the bash shell, using the BLAST manual as a guideline. The BLAST program makeblastdb was run to set up the database. The blastdb_aliastool was then run to compile each database. The BLASTP analysis was performed using a small bash script, using non-default parameters, qcov 0.6 for the coverage, e-value was set at 1 × 10^5^, and the output format was set to ‘6’, which gives a tabular format to the BLAST results.

### 4.3. Post-BLAST Processing

Following the BLAST analysis, another filtering algorithm was applied to identify the hits with at least 40% identity. Of the alignments that passed this identity filter, we aimed to identify only the best alignment from each of the queries (alignment with the highest coverage). That is, even if a query aligns with more than one subject within the same family, or aligns with more than one family, only the best one is considered. 

Each alignment was then divided into three categories based on its genomic location: (i) exclusively on the plasmid; (ii) exclusively on the chromosome; or (iii) both (on the plasmid and on the chromosome). There are no overlapping counts; this means that if a given genome has one or more copies of genes of a certain category on a plasmid and chromosome, the ‘both’ category count will be incremented by one and will not increase the counts on any of the individual plasmid or chromosome category. To perform this division, we used the feature tables for each genome that are available on RefSeq.

### 4.4. Normalization to the Size of the Genomic Location

Chromosomes and plasmids differ in size, which could impact the probability of encoding ARs or VFs. To avoid this effect, we also counted, for each genome, how many CDS exist for each genomic location. Subsequently, the counts of VF and AR orthologues per genomic location were divided by the number of CDS in that location.

### 4.5. Graphical and Statistical Analysis

The bar plots and the boxplots were created using the R ggplot2 package [43]; the linear regression models were created using R [44]; the heatmaps were created using the R gplots package [45], and the Kruskal–Wallis tests were performed with R [44].

### 4.6. Observed/Expected Ratio Calculation

To calculate the extent to which each AR orthologue and each VF orthologue co-localized more often or less often than expected, we proceeded as follows. First, we built a matrix where, in each position, we had the number of times each AR orthologue and each VF co-localizes (in the chromosome of the same bacterial cell, in plasmids of the same bacterial cell, or in both). Then, we calculated another matrix, but with the expected values. These expected values were calculated for ResFinder, MUSTARD, and VFDB orthologues, from the observed count of orthologues per category multiplied by the total number of orthologues in that genomic location (of all categories) divided by the total number of orthologues found pertaining to the respective database. From this table, we constructed another matrix, for each database, with the observed/expected ratios of antibiotic resistance gene orthologues in each database category per genomic location.

The details to build the matrix with the expected values are as follows. Firstly, for each observation, the expected value was calculated using

(1) $$E\left(AR_{i},\;VF_{j}\right)=\frac{\sum_{k}Obs\left(AR_{i},VF_{k}\right)\cdot \sum_{m}Obs\left(AR_{m},VF_{i}\right)}{\sum_{k}\sum_{m}Obs\left(AR_{m},VF_{k}\right)}$$

Secondly, each observation was divided by the expected value. The (*i*, *j*) position of the resulting matrix is

(2) $$\frac{Obs(AR_{i},\;VF_{j})}{E(AR_{i},\;VF_{j})}$$

These calculations were performed for each genomic position and for the co-occurrences between Resfinder and VFDB and between MUSTARD and VFDB. The third step was to calculate the ${\mathrm{Log}}_{2}\left[\frac{Obs\left(AR_{i},VF_{j}\right)}{E\left(AR_{i},VF_{j}\right)}\right]$ for each (*i*, *j*) pair. The final tables were used to create the heatmaps. We considered relevant those cases where the number of observations was less than half of the expected value, so *Obs*/*E* < ½ (therefore, ${\mathrm{Log}}_{2}\left[\frac{Obs}{E}\right]$ is less than Log_2_(½) = −1) or where the number of observations was higher than double the expected value *Obs*/*E* > 2 (therefore, ${\mathrm{Log}}_{2}\left[\frac{Obs}{E}\right]>{\mathrm{Log}}_{2}\left[2\right]=1$.

If *Obs*(*AR_i_*,*VF_j_*) = 0 and *E*(*AR_i_*,*VF_j_*) > 0, *Obs*(*AR_i_*,*VF_j_*)/*E*(*AR_i_*,*VF_j_*) = 0. For these cases, and because Log(0) = −infinity, we changed the zero to the lowest number of the table divided by 10. Furthermore, we did not include the (*AR_i_*,*VF_j_*) cases in the heatmaps when *Obs*(*AR_i_*,*VF_j_*) = 0 and *E*(*AR_i_*,*VF_j_*) = 0.

## Data Availability

Not applicable.
